# The effects of bupropion alone and combined with naltrexone on weight loss: a systematic review and meta-regression analysis of randomized controlled trials

**DOI:** 10.1186/s13098-024-01319-7

**Published:** 2024-04-24

**Authors:** Yang Liu, Fei Han, Zefeng Xia, Ping Sun, Pejman Rohani, Palanisamy Amirthalingam, Mohammad Hassan Sohouli

**Affiliations:** 1 Department of Gastrointestinal Surgery, Union Hospital, Tongji Medical College, Huazhong University of Science and Technology, 430022 Wuhan, China; 2 Department of Hepatobiliary Surgery, Union Hospital, Tongji Medical College, Huazhong University of Science and Technology, 1277 Jiefang Avenue, 430022 Wuhan, China; 3 Pediatric Gastroenterology and Hepatology Research Center, Pediatrics Centre of Excellence, Children’s Medical Center, Tehran University of Medical Sciences, Tehran, Iran; 4 Department of Pharmacy Practice, Faculty of Pharmacy, University of Tabuk, Tabuk, Saudi Arabia; 5 Student Research Committee, Department of Clinical Nutrition and Dietetics, Faculty of Nutrition and Food Technology, Shahid Beheshti University of Medical Sciences, Tehran, Iran

**Keywords:** Bupropion, Naltrexone, Obesity, Weight loss, Meta-analysis

## Abstract

**Background:**

The global prevalence of obesity and overweight is a significant concern in the field of public health. However, addressing and combating these conditions pose considerable challenges. Numerous interventional studies have been conducted to assess the possible impact of bupropion on weight reduction. The primary objective of this study was to conduct a comprehensive investigation into the effects of bupropiona alone and in combination with naltrexone on weight, body mass index (BMI), and waist circumferences (WC).

**Methods:**

A systematic search was conducted in five databases using established keywords. The purpose of this search was to uncover controlled trials that examined the impact of bupropion, either as a standalone intervention or in combination with naltrexone, on weight loss outcomes. The random-effects model analysis was used to provide pooled weighted mean difference and 95% confidence intervals.

**Results:**

Twenty five studies with 22,165 participants’ were included in this article. The pooled findings showed that bupropion administration has an effect on lowering weight (WMD: -3.67 kg, 95% CI: -4.43 to -2.93) and WC (WMD: -2.98 cm, 95% CI -3.78 to -2.19) in compared with control groups. The analysis also showed that the effects of the present intervention on weight and WC during the intervention are > 26 weeks and ≤ 26 weeks compared to the other group, respectively. In addition, changes in weight loss and WC after receiving bupropion together with naltrexone were more compared to bupropion alone.

**Conclusions:**

In conclusion, the addition of combination therapies like bupropion and naltrexone to lifestyle modifications including diet would cause significant weight loss.

**Supplementary Information:**

The online version contains supplementary material available at 10.1186/s13098-024-01319-7.

## Introduction

Obesity is a prevalently increasing chronic disease and a global epidemic [1]. According to the World Health Organization, BMI > 25 kg/m^2^ is considered overweight and BMI > 30 kg/m^2^ is considered obese. These conditions occur due to abnormal and excessive accumulation of fat, which is related to high health hazards and chronic diseases [2]. Obese or overweight individuals are at high risk of various comorbidities like hypertension, diabetes mellitus and cardiovascular disease. Moreover, they are at high mortality risk [3]. Most of these complications caused by obesity may lead to death, which can be altered by lifestyle modifications. Different mechanisms like insulin resistance, inflammation, abnormalities in the metabolism of lipid, adipokine balance and endothelial function have been proposed [4]. Moreover, minimal grade chronic inflammation induced by high pro-inflammatory cytokines and adipokines secretion by macrophages and adipocytes of adipose tissue have been proposed as a mechanism for obesity [5]. Many first-generation and second-generation antipsychotics block 5HT2c serotonin receptors and cause weight gain, in which 5HT2c serotonin receptors are involved in regulation of appetite [6]. Even with modest reduction of weight, obesity-related diseases and obesity-related risk factors can be reduced [7].

In the Unites States, bupropion, a norepinephrine/dopamine-reuptake inhibitor (NDRI) was approved for depression in 1990s [8]. Studies have reported that bupropion during the treatment of depression, act as antidepressant and also cause weight changes. Some studies proposed that bupropion causes significant weight gain [9, 10], some studies proposed that it cause significant weight loss [11, 12] and some stated no significant change in weight [13, 14]. A systematic review and meta-analysis collected evidence about bupropion and reported that bupropion does not cause weight gain and it mainly induces weight loss [15].

Naltrexone is an opioid antagonist, FDA approved for use to treat alcohol use disorder [16]. It was reported that opioid antagonist block central opioid receptors and thus reduce the intake of certain foods [17]. Naltrexone through the action of β-endorphin blockage at the receptor of µ-opioid would reduce the consumption of food [18].

The combination effect of bupropion and naltrexone on weight loss is not precise. It was proposed theoretically that naltrexone could influence the neurological pathways of the brain, while bupropion would suppress the appetite [19] Two studies have reported that bupropion/naltrexone combination was found to be effective in achieving loss of weight in obese individuals [18, 20]. Bupropion/naltrexone combination produced significant weight loss when compared to either medication alone. The mechanisms through which naltrexone/bupropion combination act in reducing weight are not completely clear. α-melanocyte-stimulating hormone and β-endorphin are released by pro-opiomelanocortin producing neurons present in the hypothalamus. α-melanocyte-stimulating hormone regulates the activation of pro-opiomelanocortin and β-endorphin activates opioid receptors on pro-opiomelanocortin neurons causing autoinhibitory feedback [21]. Bupropion monotherapy results in modest loss of weight by increasing the pro-opiomelanocortin firing [22]. The weight loss caused by bupropion might be achieved by β-endorphin-mediated autoinhibitory feedback loop and adding naltrexone to bupropion would prevent this negative feedback loop and may help in sustained reduction of weight and achieving weight loss.

The purpose of the current systematic review and meta-regression analysis of randomized controlled trials is to present the evidence systematically on the effects of bupropion alone and combined with naltrexone on weight, body mass index (BMI), waist circumferences (WC).

## Methods

### Search strategy

This study adhered to the guidelines outlined by the Preferred Reporting Items for Systematic Review and Meta-analysis (PRISMA) criteria [23]. A comprehensive search was conducted in the PubMed/MEDLINE, Web of Science, SCOPUS, and Embase databases, without any limitations on language or time, covering the period up to February 2023. Furthermore, the search encompassed relevant scholarly articles as well as gray literature. The selection of Medical Subject Headings (MeSH) and Emtree (Embase Subject Headings) was made in order to conduct searches on the online databases: (“Bupropion” OR " naltrexone " OR " Amfebutamone " OR " Wellbutrin " OR “Zyban " OR “Quomen” OR “Zyntabac “) AND (“weight” OR “Waist Circumference” OR “Body Mass Index”) AND (“Clinical Trials as Topic” OR “Cross-Over Studies” OR “Double-Blind Method” OR “Single-Blind Method” OR “Random Allocation” OR “Clinical Trial”). The reference lists of the publications that were obtained and the review papers that were linked were examined manually in order to find any qualifying trials that may have been overlooked.

### Eligibility criteria

The process involved two researchers independently eliminating duplicate articles based on titles, abstracts, or the whole texts of the research, followed by the identification and evaluation of pertinent publications. Ultimately, the papers were categorized according to the aforementioned criteria: (1) The study employed a randomized clinical trial design. (2) The intervention involved administering bupropion alone and in combination with naltrexone to individuals aged 18 and older. (3) Baseline and post-intervention measurements of weight, body mass index (BMI), and waist circumference (WC) were collected for both the intervention and control groups. When a research study reported outcomes at multiple follow-up times, the data from the most recent or longest follow-up period was utilized. Excluded from the analysis were studies that contained duplicated data, studies that specifically examined the impact of bupropion on smoking cessation, studies that provided unclear or ambiguous information, studies that utilized bupropion as an intervention in conjunction with other frequently prescribed medications (excluding naltrexone), studies that employed non-randomized trial designs, studies conducted on animals, studies lacking a control group, as well as reviews and meta-analyses. The criteria for inclusion and exclusion of studies, known as the PICOS criteria, were as follows. The study population included of individuals who were 18 years of age or older. The intervention being investigated was the administration of bupropion either alone or in combination with naltrexone. The comparator group included individuals who received a different intervention or a placebo. The variables of interest in this study are weight, body mass index (BMI), and waist circumference (WC). The study design employed in this research investigation is that of randomized clinical trials.

### Data extraction

The authors conducted an independent examination of the qualifying studies. The study extracted various key details, including the first author’s name, the study’s location, the publication year, the sample size for both the intervention and control groups, participant characteristics (such as the percentage of men, BMI, age, and health status), the type of outcomes assessed, the duration of the intervention, the dosage and type of intervention administered, as well as the means and standard deviations (S.D.s) of the intended outcomes at baseline, post-intervention, and/or changes between baseline and post-intervention.

### Quality assessment

The evaluation of the study’s quality is detailed in Table 1. The methodological evaluation of the included randomized controlled trials (RCTs) was conducted using version 2 of the Cochrane risk-of-bias tool for randomized trials (RoB 2) [24]. The authors of this study conducted an assessment of potential sources of bias, including blinding of outcome assessment, allocation concealment, participant and staff blinding, random sequence generation, incomplete outcome data, selective reporting, and other bias. Each study was independently rated by two authors, who categorized the risk of bias as low, high, or unclear. In order to reach a consensus, any discrepancies were deliberated over with the involvement of a third author. The current analytic research was assessed for quality using the GRADE (Grading of Recommendations Assessment, Development, and Evaluation) grading technique. The GRADE checklist is a robust 10-point grading method that evaluates factors that impact the quality of a study. The scale comprises seven distinct components, namely: (1) risk of bias (2) precision, (3) heterogeneity, (4) directness, (5) publication bias, (6) financing bias, and (7) study design [25].

**Table 1:** Risk of bias assessment according to the Cochrane collaboration’s risk of bias assessment tool

Study, Year (reference)	Random sequence generation	Allocation concealment	Blinding of participants and personnel	Blinding of outcome assessment	Incomplete outcome data	Selective reporting	Overall assessment of risk of bias
Weizman et al. 2021	Low	Low	Low	Low	Unclear	Low	Low
McIntyre et al. 2021	Low	Unclear	Low	Low	Unclear	Low	Unclear
Wadden et al. 2011	Low	Low	Low	High	Unclear	Low	Unclear
Jain et al. 2002	Low	Low	Low	Low	Unclear	Low	Low
Hong et al. 2016	Low	Unclear	Low	Low	Unclear	Low	Unclear
Cho et al. 2020	Low	Low	Low	Low	Unclear	Low	Low
Hollander et al. 2013	Low	High	Low	Low	Unclear	Low	Low
Halseth et al. 2016	Low	Low	High	Low	Unclear	Low	Unclear
Lyu et al. 2018	Low	Low	Unclear	Low	Unclear	Low	Low
Bajaj et al. 2020	Low	Unclear	Low	Low	Unclear	Low	Unclear
Greenway et al. 2009	Low	Low	Low	Low	Unclear	Low	Low
Greenway et al. 2010	Low	Unclear	Unclear	Low	Unclear	Low	Low
Apovian et al. 2013	Low	High	High	Low	High	Low	High
Nissen et al. 2016	Low	Low	High	Unclear	Unclear	Low	Unclear
Gadde et al. 2001	Low	Low	Unclear	Unclear	Unclear	Low	Unclear
Smith et al. 2013	Low	Low	High	Low	Unclear	Low	Unclear
Croft et al. 2002	Low	Low	Unclear	Low	Unclear	Low	Low
Grilo et al. 2022	Low	Low	Unclear	Unclear	Unclear	Low	Unclear
Anderson et al. 2002	Low	Low	High	Low	Unclear	Low	Unclear
Wharton et al. 2021	Low	Low	Unclear	Low	Unclear	Low	Low
Settle et al. 1999	Low	Unclear	Low	Low	Unclear	Low	Unclear
Weihs et al. 2002	Low	Low	Unclear	Unclear	Unclear	Low	Unclear
Grilo et al. 2020	Low	Low	Low	Low	Unclear	Low	Low
White et al. 2013	Low	High	Low	Low	Unclear	Low	Low
Billes et al. 2011	Low	Low	Low	Low	Unclear	Low	Low

### Data synthesis and statistical analysis

The data were analyzed using the STATA version 12.0 program. Furthermore, the utilization of Endnote software facilitated the elimination of duplicate articles and the effective management of eligible articles. Various data types were subjected to a predetermined technique for conversion, resulting in the calculation of their mean and standard deviations (S.D.s) as reported in references [26, 27]. In the event that standard deviations are not available, the change was computed utilizing the following method: The formula for calculating the change in standard deviation is derived as the square root of the difference between the sum of the squares of the baseline standard deviation and the final standard deviation, subtracted by twice the product of the baseline standard deviation and the final standard deviation correlation coefficient. The formula utilized for converting the standard error of the mean (SEM) to the standard deviation is as follows: The standard deviation (S.D.) can be calculated by multiplying the standard error of the mean (SEM) by the square root of the total number of participants (n) in each group. The utilization of the random-effects model was employed in the meta-analysis of the research findings. The research weighted according to the conventional inverse variance methodology. The analysis utilized the data obtained from the longest time point, enabling the inclusion of many examinations within a single study group. The evaluation of study heterogeneity was conducted using Q Statistics and I-squared (I^2^). The study identified varying levels of heterogeneity, categorized as insignificant, low, moderate, and high. These levels were quantified using I^2^ values, which ranged from 0 to 25%, 26–50%, 5–75%, and 76–100%, respectively [28]. In order to ascertain potential factors contributing to heterogeneity, a predetermined subgroup analysis was performed, taking into account the dosage, duration, and kind of intervention. A sensitivity analysis was conducted in order to ascertain the individual contributions of each research study to the overall mean difference. To assess the presence of publication bias, we employed the Egger’s test, a widely recognized statistical method [29].

## Results

**Fig. 1:**
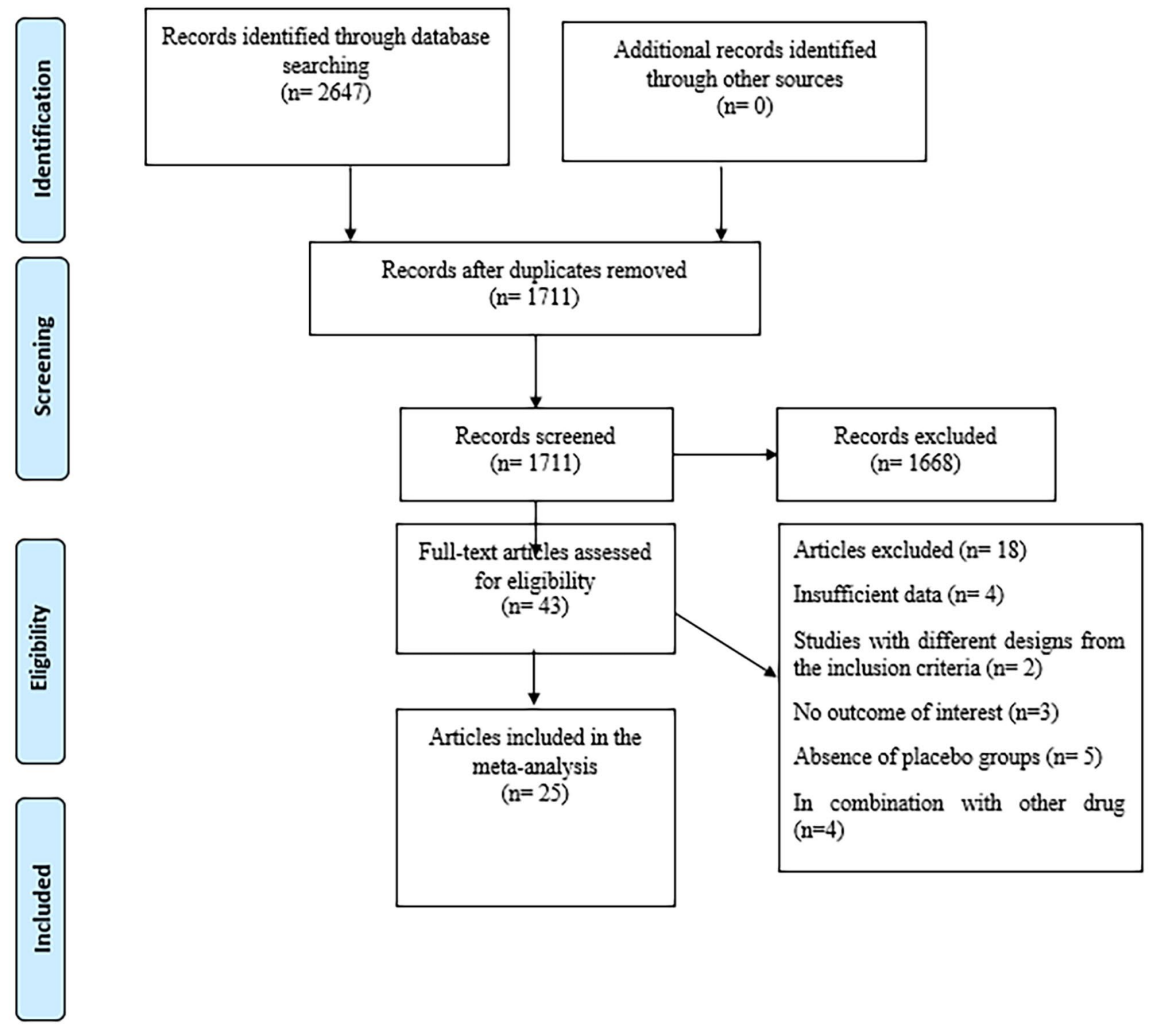
Flow chart of the study, including identification, screening, eligibility, and the final sample included

The flowchart presented in Fig. 1 illustrates the research selection procedure, incorporating exclusion criteria. The aforementioned electronic databases yielded a total of 2647 articles. Following the elimination of redundant research papers, the cumulative count amounted to 1711. After conducting an evaluation of the titles and abstracts of the research papers, a total of 1668 articles were excluded from the study due to their failure to match the specified inclusion criteria. A total of 43 publications were identified through the implementation of a comprehensive full-text search method throughout the secondary screening process. Eighteen investigations were discontinued based on the aforementioned factors. A total of 25 publications, each containing 33 treatment arms, were deemed eligible for inclusion in the quantitative meta-analysis based on their adherence to the specified qualifying criteria.

**Fig. 2:**
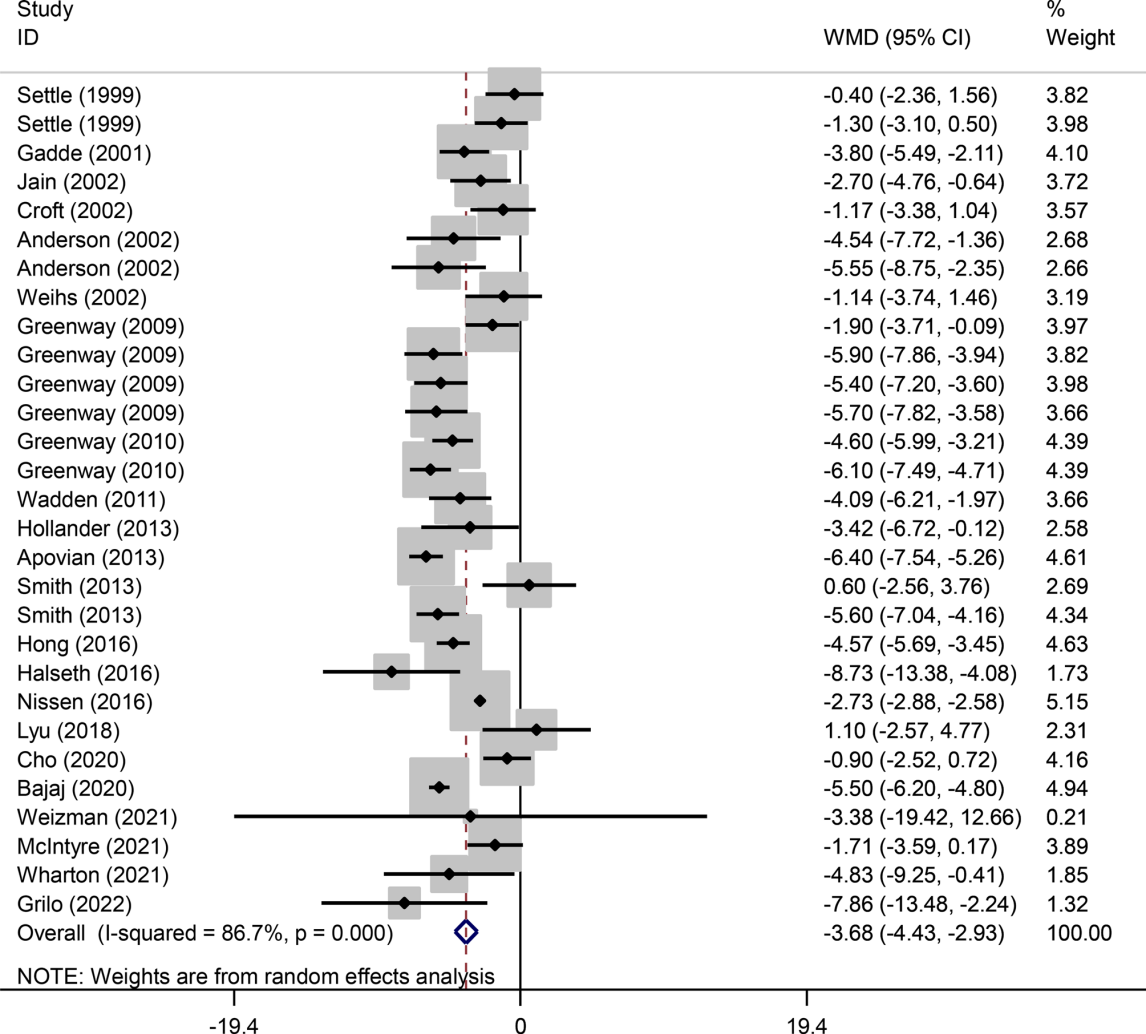
Forest plot of randomized controlled trials investigating the effects of bupropion alone and combined with naltrexone on weight (kg)

**Fig. 3:**
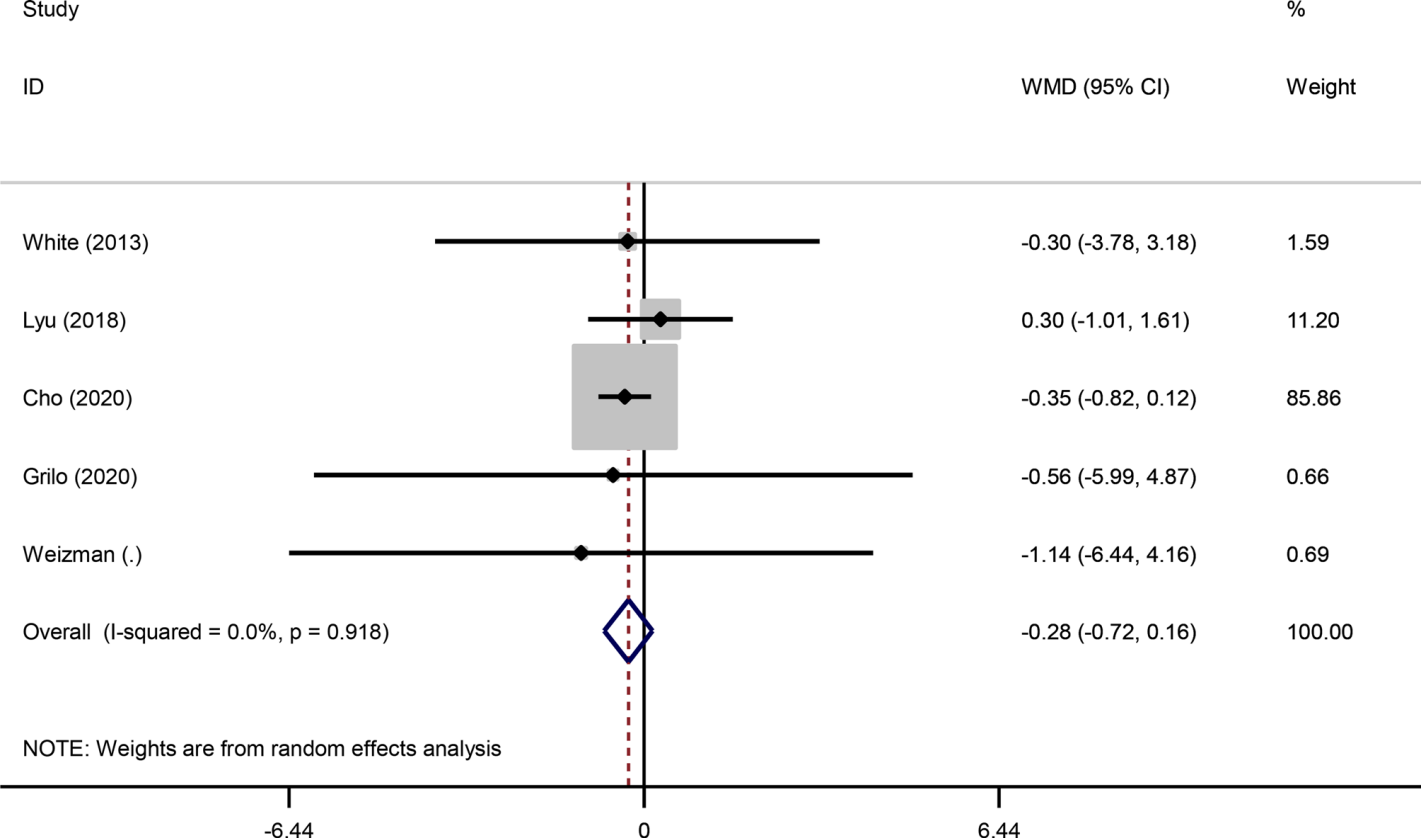
Forest plot of randomized controlled trials investigating the effects of bupropion alone and combined with naltrexone on body mass index (BMI) (kg/m^2^)

### Study characteristics

The characteristics of the aggregated articles are presented in Table 2. According to our survey findings, a total of twenty studies have been done in the United States, while three papers have been published in Canada. Additionally, one study each has been carried out in Israel and the Republic of Korea, respectively. The publications included in this study were published between the time frame of 1999 to 2021, and the duration of the follow-up interventions varied from 8 to 104 weeks. At the baseline, the average age and proportion of male participants varied between 35.2 and 61.6 years and 0-100%, respectively. The doses prescribed in the studies were between 90 and 400 mg per day, and Bupropion alone was used in 7 studies and the combination of this drug with naltrexone was used as the intervention group in the rest of the studies. The mean BMI at the baseline level was between 26.1 and 37.1 in the studies included. In 14 studies, the intervention was carried out on overweight and obese people. In 8 articles, obese people with psychiatric problems and eating disorders were considered as the study population, and the remaining 3 studies were conducted on diabetics and breast cancer patients.

**Table 2:** Characteristics of eligible studies

Author (year)	Country	Population	Mean Age year	Sex (Male %)	Sample Size Study (intervention)	Sample Size Study (control)	Follow up of intervention (Weeks)	Type and dose (mg/day) of intervention	Baseline of BMI (kg/m^2^)	Outcomes	
Weizman et al. 2021	Israel	Individuals With Schizophrenia	35.2	41.6	12	10	8	Bupropion SR (150–300 mg/d)	35	Weight, BMI	
McIntyre et al. 2021	Canada	Patients with Obesity or Overweight	61.6	53	890	585	104	Naltrexone SR( 32 mg/day) with bupropion SR (360 mg/day)	36.9	Weight	
Wadden et al. 2011	USA	Patients with Obesity or Overweight	45.9	10.7	591	202	56	Naltrexone SR ( 32 mg/day) with bupropion SR (360 mg/day)	36.3	Weight, WC	
Jain et al. 2002	USA	Obese Patients with Depressive Symptoms	46	11	213	209	26	Bupropion SR (300 mg/day)	36	Weight	
Hong et al. 2016	USA	Patients with Obesity or Overweight	44.8	17.1	1489	1176	56	Naltrexone SR (32 mg/day) with bupropion SR (360 mg/day)	36.2	Weight	
Cho et al. 2020	Republic of Korea	Obese Breast Cancer Survivors	56	0	14	20	8	Naltrexone (8 mg/day) with bupropion (90 mg/day)	27.6	Weight, BMI	
Hollander et al. 2013	USA	Overweight/obese individuals with type 2 diabetes	54	46	265	159	56	Naltrexone SR ( 32 mg/day) with bupropion SR (360 mg/day)	37	Weight, WC	
Halseth et al. 2016	USA	Obesity	46.1	18.3	71	82	26	Naltrexone SR (32 mg/day) with bupropion SR (360 mg/day)	36.33	Weight, WC	
Lyu et al. 2018	USA	Obese male schizophrenia patients	54.3	100	11	10	24	Naltrexone SR ( 24 mg/day) with bupropion SR (300 mg/day)	26.1	Weight, BMI	
Bajaj et al. 2020	Canada	Adults with overweight or obesity	NR	21	1310	763	56	Naltrexone SR ( 32 mg/day) with bupropion SR (360 mg/day)	36.2	Weight	
Greenway et al. 2009	USA	Obesity	42.6	13.3	36,32, 38, 18	84	24	Bupropion SR (400 mg/d), naltrexone SR (16, 32, or 48 mg/day) with bupropion SR (400 mg/day)	35.1	Weight, WC	
Greenway et al. 2010	USA	Overweight and obese adults	44.4	11.5	471, 471	511	56	Naltrexone SR (16 mg/day) wit bupropion SR (360 mg/day)	36.1	Weight, WC	
Apovian et al. 2013	USA	Overweight and obese participants	44.3	15	434	267	56	Naltrexone SR (32 mg/day) with bupropion SR (360 mg/day)	36.2	Weight, WC	
Nissen et al. 2016	USA	Overweight and Obese Patients With Cardiovascular Risk Factors	61	15	4455	4450	104	Naltrexone SR (32 mg/day) with bupropion SR (360 mg/day)	36.6	Weight, WC	
Gadde et al. 2001	USA	Patients with Obesity or Overweight women	40.4	15.4	25	25	8	Naltrexone SR (32 mg/day) with bupropion SR (360 mg/day)	36.9	Weight	
Smith et al. 2013	USA	Obese subjects	46	45.5	8, 41	19	24	Bupropion SR (400 mg/d)/ Naltrexone SR (48 mg/day) with bupropion SR (400 mg/day)	358	Weight, WC	
Croft et al. 2002	USA	Patients with Major Depression	39.4	NR	210	213	52	Bupropion SR (300 mg/d)	28.74	Weight	
Grilo et al. 2022	USA	Binge-Eating Disorder	46.5	18.4	32	34	16	Naltrexone SR (32 mg/day) with bupropion SR (360 mg/day)	37.1	Weight	
Anderson et al. 2002	USA	Obese adults	43.9	16.3	110, 105	112	48	Bupropion SR (300 or 400 mg/d)	36.5	Weight, WC	
Wharton et al. 2021	Canada	Subjects with type 2 diabetes	60.7	44.9	165	96	52	Naltrexone SR (32 mg/day) with bupropion SR (360 mg/day)	37.5	Weight	
Settle et al. 1999	USA	Depressed outpatients.	38.9	35	113, 111	347	8	Bupropion SR (200 or 400 mg/d)	NR	Weight	
Weihs et al. 2002	USA	Patients with recurrent major depression	39.4	34	210	213	8	Bupropion SR (300 mg/d)	NR	Weight	
Grilo et al. 2020	USA	Binge-eating Disorder with Obesity	50.4	13.6	9	7	12	Naltrexone SR (50 mg/day) with bupropion SR (300 mg/day)	27.6	BMI	
White et al. 2013	USA	Overweight and obese women	44.15	0	31	30	8	Bupropion SR (300 mg/d)	35.8	BMI	
Billes et al. 2011	USA	Obese subjects	NR	NR	273	277	56	Naltrexone SR (16 or 32 mg/day) wit bupropion SR (360 mg/day)	NR	WC	

BMI: body mass index; WC: waist circumferences; NR: not report; SR: sustained-release

Table 1 displays the results of the assessment conducted to evaluate the quality of the eligible studies. Moreover, the quality of the present meta-analysis was evaluated using the GRADE score method, resulting in a grade of 8.4, indicating a very good level of quality.

### Meta-analysis results

**Fig. 4:**
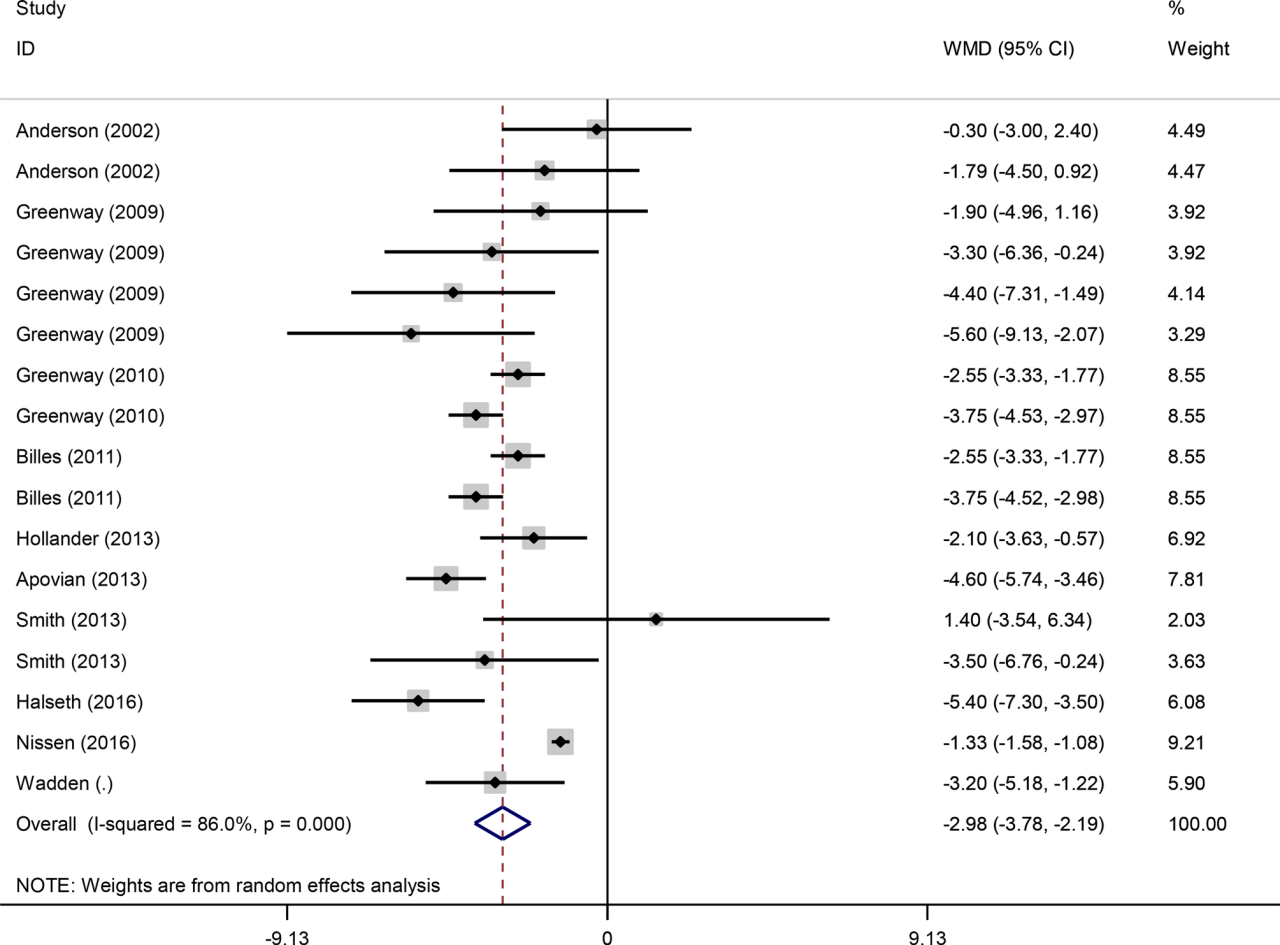
Forest plot of randomized controlled trials investigating the effects of bupropion alone and combined with naltrexone on waist circumference (WC) (cm)

Pooled findings from the random-effects model indicated that body weight (weighted mean difference (WMD): -3.67 kg, 95% confidence interval (CI): -4.43 to -2.93, *P* < 0.001) and waist circumference (WC) (WMD: -2.98 cm, 95% CI -3.78 to -2.19, *P* < 0.001) were significantly reduced after bupropion alone and combined with naltrexone compared to control group. However, no significant effect was observed on body mass index (BMI) (WMD: -0.28 kg/m^2^, 95% CI: -0.72 to 0.16, *P* = 0.207) compared to the control group. Furthermore, significant heterogeneity was found among the studies for weight (Cochran *Q* test, *P* < 0.001, I^2^ = 86.7%) and WC (Cochran *Q* test, *P* < 0.001, I^2^ = 86.0%). However, low heterogeneity was reported for BMI (Cochran Q test, *P* = 0.918, I^2^ = 0.0%) (Figs. 4, 2 and 3).

The subgroup analysis also showed that the effects of the present intervention on weight and WC during the intervention are greater than 26 weeks and less equal to 26 weeks compared to the other group, respectively. In addition, the subgroup analysis showed that changes in weight loss and WC after receiving bupropion together with naltrexone were more compared to bupropion alone (Supplementary Table).

### Meta-regression

Meta-regression between bupropion and absolute mean differences in body weight and WC based on dosage and duration of intervention was performed. There was a significant relationship between dose of intervention with changes in body weight (coefficient (Coef) = -0.0146719, *P* = 0.012). However, meta-regression analysis not showed a significant linear relationship between dose and duration of intervention with changes in WC (Supplementary Figs. 1–2).

### Sensitivity analysis

In order to ascertain the impact of each individual article on the overall effect size for weight, BMI, and WC, we systematically removed each experiment from the analysis in a sequential manner. The sensitivity analysis, namely the leave-one-out approach, demonstrated the robustness of the obtained results (Supplementary Fig. 3).

### Publication bias

Upon visual examination of the funnel plot, no indications of publication bias were observed. This finding was further supported by the results of the Egger’s tests, which revealed no evidence of publication bias for weight (*P* = 0.183), BMI (*P* = 1.00), and WC (*P* = 0.127; Supplementary Fig. 4).

## Discussion

Overweight and obesity leads to increased cardiovascular disease risk and mortality [30]. Hence it is essential to lose weight in order to reduce the risk of cardiovascular events. This systematic review and meta-regression analysis of randomized controlled trials reported that body weight and waist circumference were significantly reduced after bupropion alone and combined with naltrexone compared to control group. Similar result was obtained in another study, which demonstrated that naltrexone plus bupropion was associated with significant weight loss and thus improving cardiovascular risk factors when compared to placebo. There was more weight loss with naltrexone plus bupropion at fourth week and it was maintained until 56 weeks. Moreover, naltrexone plus bupropion caused reduction in waist circumference [31]. In our study, the subgroup analysis revealed that there was significant weight loss at duration > 26 weeks and ≤ 26 weeks, but the weight loss was more in duration > 26 weeks. Regarding waist circumference, significant reduction of waist circumference was observed at duration > 26 weeks. In addition, our subgroup analysis showed that changes in weight loss and waist circumference after receiving bupropion together with naltrexone were more compared to bupropion alone. As monotherapy, bupropion is used for treating nicotine dependence and naltrexone for alcohol and opioid dependence. The efficacy of bupropion and naltrexone in the treatment of addiction disorders may potentially enhance individuals’ capacity to regulate their eating behaviors and mitigate food cravings to some extent [32].

In our systematic review and meta-analysis, it was observed that the weight loss was more with bupropion combined with naltrexone when compared with bupropion alone. This result was similar to another study [20]. More than additive effects, bupropion combined with naltrexone produces synergistic effect in reducing body weight [18]. Greater weight loss with duration > 26 weeks in our study may be due to the fact that endorphin-mediated autoinhibition of hypothalamic pro-opiomelanocortin neurons are prevented by naltrexone which enhances the effectiveness of stimulation of melanocortin when combined with bupropion [18]. The mechanism of hypothalamus can cause weight loss for longer duration in subjects treated with bupropion/naltrexone combination.

Our subgroup analysis showed that changes in waist circumference after receiving bupropion together with naltrexone were more compared to bupropion alone. This result was consistent with another study [18, 20].

In our meta-analysis, no significant effect was observed on body mass index with bupropion/naltrexone combination compared to the control group. This is in contrast with other study, which reported that there was a significant decrease in waist circumference in bupropion/naltrexone group compared to placebo [33]. The contrasting result may be due to the fact that the study was conducted in overweight and obese patients with cardiovascular risk factors.

A meta-regression analysis was conducted to examine the relationship between bupropion and the absolute mean differences in body weight, BMI, and waist circumference, taking into account the dosage and duration of the intervention. A notable correlation was observed between the levels of intervention administered and the alterations in body weight. However, meta-regression analysis did not show a significant linear relationship between dose and duration of intervention with changes in waist circumference. Similar to our study results was obtained in another study [34]. Historical data has suggested that it is difficult for a person to maintain weight loss but it is easy to achieve it at the beginning [35]. This data is supported by a meta-analysis, which reported that 50% of lost weight was regained in 2 years and 80% of lost weight was regained in 5 years [36]. Evidence based on recent data suggested that it is possible for majority of the people who lose their weight, to maintain their weight for a longer duration [37].

Animal studies also have proved that administration of bupropion or naltrexone caused dose-dependent decrease in intake of food in mice [38] and rats [39]. Combination of bupropion and naltrexone caused much greater decreased in intake of food, producing synergistic effect more than additive effect [39].

### Strengths and limitations

Our systematic review and meta-regression analysis of randomized controlled trials evaluated the effects of bupropion alone and combined with naltrexone on weight loss taking into account the body weight, BMI and waist circumference. Moreover, dose and duration of intervention was also analyzed.

The main limitation is that adverse effects of long term effects of bupropion combined with naltrexone was not taken into account. The weight loss in normal weight persons, overweight and obese persons were not assessed individually. The consequences of non-adherence to the medications were not assessed. Significant heterogeneities were identified both clinically and statistically. The observed variations can be attributed to distinct intervention-specific factors, such as the specific type of regimen, dosage of supplements, and duration of protocols, as well as patient-specific factors, including genetic makeup, age, gender, ethnicity, medical history (including any prior instances of the disease), use of medications or supplements, and allergies to substances.

## Conclusion

In conclusion, the addition of combination therapies like bupropion and naltrexone to lifestyle modifications including diet would cause significant weight loss, which would have an impact on obesity-related complications. Bupropion combined with naltrexone would be used to treat obesity.

### Electronic supplementary material

Below is the link to the electronic supplementary material.

Supplementary Material 1

Supplementary Material 2

## Data Availability

Data is available upon request from the corresponding author for the article due to privacy / ethical restrictions.
