# Supplementary material for: The effects of bupropion alone and combined with naltrexone on weight loss: a systematic review and meta-regression analysis of randomized controlled trials
Source: Diabetol Metab Syndr. 2024 Apr 24;16:93. doi: 10.1186/s13098-024-01319-7 (PMC11044307; doi:10.1186/s13098-024-01319-7)
Supplement: Supplementary file 1 — Supplementary Material 1 [file 13098_2024_1319_MOESM1_ESM.docx]

**Supplemental Table 1-** Meta-analyses showing the effect of bupropion alone and combined with naltrexone on several subgroups (all analyses were conducted using random effects model).

|  | | | | **Heterogeneity** |  |  |
| --- | --- | --- | --- | --- | --- | --- |
|  | | **No. of**  **Treatment arms** | **WMD^1^ (95%CI)** | | ***I^2^* (%)** | ***P* within group** |
| **Weight (kg)** | |  |  | |  |  |
| ***Duration*** | *≤ 26 weeks* | 16 | **-3.12 -4.39 -1.86** | | ***79.4*** | ***<0.001*** |
|  | *> 26 weeks* | 13 | **-4.27 -5.37 -3.17** | | ***91.2*** | ***<0.001*** |
| ***Dosage*** | *≤ 360 mg* | 20 | **-3.55 -4.49 -2.61** | | ***88.3*** | ***<0.001*** |
|  | *>360 mg* | 9 | **-3.92 -5.32 -2.53** | | ***77.7*** | ***<0.001*** |
| **Type of intervention** | *bupropion alone* | 11 | **-2.10 -3.10 -1.11** | | ***46.5*** | ***0.045*** |
|  | *bupropion combined with naltrexone* | 18 | **-4.50 -5.4.9 -3.51** | | ***90.8*** | ***<0.001*** |
| **BMI (kg/m^2^)** | |  |  | |  |  |
| **Type of intervention** | *bupropion alone* | 2 | **-0.55 -3.46 2.36** | | ***0.0*** | ***0.79*** |
|  | *bupropion combined with naltrexone* | 3 | **-0.28 -0.72 0.17** | | ***0.0*** | ***0.65*** |
| **Waist circumference ( cm)** | |  |  | |  |  |
| ***Duration*** | *≤ 26 weeks* | 7 | **-3.73 -5.19 -2.26** | | ***37.8*** | ***0.14*** |
|  | *> 26 weeks* | 10 | **-2.73 -3.63 -1.83** | | ***90.3*** | ***<0.001*** |
| ***dosage*** | *≤ 360 mg* | 10 | **-3.00 -3.93 -2.06** | | ***91.4*** | ***<0.001*** |
|  | *>360 mg* | 7 | **-2.97 -4.33 -1.62** | | ***19.0*** | ***0.285*** |
| **Type of intervention** | *bupropion alone* | 4 | **-1.02 -2.56 0.52** | | ***86.0*** | ***<0.001*** |
|  | *bupropion combined with naltrexone* | 13 | **-3.35 -4.23 -2.47** | | ***89.2*** | ***<0.001*** |
