# Supplementary material for: The effects of bupropion alone and combined with naltrexone on weight loss: a systematic review and meta-regression analysis of randomized controlled trials
Source: Diabetol Metab Syndr. 2024 Apr 24;16:93. doi: 10.1186/s13098-024-01319-7 (PMC11044307; doi:10.1186/s13098-024-01319-7)
Supplement: Supplementary file 2 — Supplementary Material 2 [file 13098_2024_1319_MOESM2_ESM.docx]

|  |  |
| --- | --- |
| **Supplementary Figures 1**. Meta-regression analysis encompassing weight changes according to the duration of intervention (weeks) and dose of intervention (mg). | |

|  |  |
| --- | --- |
| **Supplementary Figures 2**. Meta-regression analysis encompassing WC changes according to the duration of intervention (weeks) and dose of intervention (mg). | |

| A)  | B)  |
| --- | --- |
| C)  |  |

**Supplementary Figures 3**. Sensitivity analysis of the weighted mean difference (WMD) for A) weight, B) BMI, and C) WC changes. *BMI*: Body mass index, *WC*: Waist circumference

| A)  | B)  |
| --- | --- |
| C)  |  |

**Supplementary Figures 4**. Funnel plots for evaluation of publication bias of A) weight, B) BMI, and C) WC. *BMI*: Body mass index, *WC*: Waist circumference
